# The Hospital. Nursing Section

**Published:** 1903-01-31

**Authors:** 


### The Hospital.

### Nursing Section.

Contributions for this Section of "The Hospital" should be addressed to the Editor, "The Hospital" Nursing Section, 28 & 29 Southampton Street, Strand, London, W.C.

No. 853.—Vol. XXXIII. *SATURDAY, JANUARY* 31, 1903.

### Notes on News from the Nursing World.

### THE FIRST MATRON OF THE HERBERT HOSPITAL.

The appointment of Miss Beatrice Isabel Jones to the post of matron at the Herbert Hospital, Woolwich, is one on which the Advisory Board of Queen Alexandra's Imperial Military Nursing Service can be warmly congratulated. Miss Jones is thoroughly experienced and admirably qualified to undertake the control of the nursing arrangements at the Herbert Hospital under the new conditions. Trained at St. Bartholomew's Hospital, where she also acted as staff nurse, she was assistant matron at Birmingham Infirmary for upwards of two years, and since 1897 she has been matron of the City of London Hospital for Diseases of the Chest. She has also had the advantage of serving in South Africa as a sister of the Army Nursing Reserve, and was one of the conspicuous successes of that body. Miss Jones has, therefore, a sufficient knowledge of the system of Army nursing, while she will also bring to bear upon the performance of her duties the practical acquaintance she has gained in a great general civil hospital, a leading workhouse infirmary, and a well-known special hospital, of all that is best in modern methods.

### CHARING CROSS NURSES' HOME.

The practical completion of the Nurses' Home at Charing Cross Hospital happily synchronises with the entrance of the new lady superintendent upon her duties. The account which we are able to give to-day of the Home shows that there is no lack of provision for the comfort and convenience of the nursing staff. There is nothing in the shape of superfluous luxuries to which subscribers to the hospital might possibly take exception, and in all directions there is evidence of careful forethought. For example, the passages have been laid with material specially prepared to deaden the sound of footsteps which might awaken sleeping nurses; the corridor which leads to the bath-rooms is additionally heated so as to prevent a sudden change from the warm bath to the cooler bedrooms; a reserve cupboard in every nurse's apartment will be found a considerable boon; and at festive gatherings the sitting-rooms, which can cleverly be thrown open, forming a continuous suite, will be greatly appreciated. There will, of course, be a formal opening ceremony, but at present the date of this event is not fixed.

### THE BRITISH NURSES' ASSOCIATION AND THE "QUALIFIED NURSE."

At a meeting of the General Council of the Royal British Nurses' Association held last Friday it was unanimously decided to enter a protest against the recognition by the Local Government Board of "women as qualified nurses who have only had one year's training in a workhouse infirmary." A letter was read from Princess Christian expressing regret that an engagement in the country would prevent her from presiding at the meeting, and assuring the council of her entire sympathy with and approval of a protest being made by the Association. Mr. Langton remarked that, although the Association might have been content with signing the petition already so widely supported, they were, he thought, a body of sufficient strength to have one of their own. The signature of the President would carry more weight than that of any other official connected with the Association. Dr. Hawkins alluded to the Departmental report as blundering and dangerous, and moved that the petition, when signed by Her Royal Highness, be sent to Mr. Walter Long. The council was unanimous, and after some remarks from Mr. Edward Fardon, who did not think the Local Government Board at all likely to adopt the suggestion as to "qualified" nurses, the meeting separated.

### A BELATED DEFENCE OF THE "QUALIFIED" NURSE.

In the opinion of the *Poor Law Officers' Journal* the proposal to create "qualified nurses" merits approval. Our contemporary's belated view is that "it should be a distinct gain to provide, as it were, an intermediate rung in the ladder whereby nurses may pass from the position of a probationer to that of a fully-trained nurse," and it refers to the proposal as tending to give promise of effective nursing in the small unions, and of providing "a link which should associate these with the larger workhouse infirmaries, where nurses may now be as fully and as competently trained as in any other hospital." But the heads of the schools which our contemporary praises, have conclusively shown that they object to the "intermediate rung in the ladder," and recognise no advantage in the provision of a link that would tend to lower the standard of nursing. As to the theory that after a nurse has received her certificate as "qualified," she will be consumed by a desire to enter into the higher ranks of the trained nurses, why should she? The very fact of training can only make her qualified, and with the guarantee of qualification which the Local Government Board are recommended to furnish in her possession, she would be far more likely to rush into private nursing and use her certificate to her own immediate pecuniary advantage. We are afraid that the *Poor Law Officers' Journal* is more concerned to render it easier for boards of guardians to obtain nurses of some kind than it is to prevent the debasement of the quality of workhouse nursing.

### A PROPOSAL TO CREATE "QUALIFIED PROBATIONERS."

THE Bath Board of Guardians have not only unanimously condemned the proposal of the Departmental Committee to create an order of Qualified Nurses, but have suggested an alternative scheme. As they are circularising all the Boards of Guardians in England and Wales with the view of inducing them to adopt a similar resolution, we give the resolutions they have passed in full. "That this Board is strongly of opinion that the term 'qualified nurse' proposed by the Departmental Committee in their report to be applied to probationers of one year's training in a minor training school is unfairly prejudicing trained nurses in general who are obliged to undergo a three years' training, as should these probationers leave the Poor Law service they are supplied with a certificate which gives them the position before the public of being fully qualified nurses. This Board, on behalf of the public generally, as well as the class of fully-trained nurses, protests against the use of this term, and is of opinion that the term 'qualified probationers' would be sufficient to meet all requirements." The objection to this suggestion is that the term probationer involves the fact of the individual being on probation for some higher post, whereas the "qualified nurse," according to the Departmental Committee's definition, has already completed her education and attained her full position, which, however, is not that of a nurse. It would be far better to call her a sick attendant.

### THE QUEEN'S NURSES IN THE HOME.

IN every respect the proceedings at the annual meeting of the Birkenhead District Nursing Society were encouraging. The number of cases visited exceeded that of 1901; the committee are in a position to take over the home occupied by the nurses and to make the necessary alterations, and the balance in hand at the end of the year exceeded £260. Moreover, testimony to the admirable work done by the nurses was given from many quarters, including that of one of the clergy who said "that he had come across them in the most hopelessly dirty homes, and he had had the opportunity of noticing the immense difference after their visit." This is a feature of the work of the district nurse which is not always recognised. She does not confine her ministrations to attendance on the sick; she seeks to render the home clean and comfortable. The importance of her efforts as an agent of sanitation, intent upon mitigating the conditions which result in disease, cannot, if they are well directed, be exaggerated.

### THE CENTRAL SICK ASYLUM.

THE report of an interview with the matron of the Central Sick Asylum at Hendon is given in another column. Miss Elma Smith was one of the numerous matrons who signed the memorial against the proposal of the Departmental Committee on Workhouse Nursing to create an order of "Qualified" nurses, whose only qualification would be one year's training. Her objection to it is the more significant as she is the head of one of the most up-to-date training schools under the Poor Law—a training school which enjoys every possible advantage, and is an admirable example of how to do it. But the good accomplished by such schools as that at Hendon would, at any rate so far as workhouse nursing is concerned, be largely undone by the adoption of the scheme which has excited the wonder and the indignation of the nursing world.

### FIRE DRILL IN NEW YORK.

THE nurses at the Bellevue Hospital, New York, are to have a new item added to their course of study. "Fire drill" is a feature of the week's work at the hospital, but hitherto the nurses have been excused from taking part in it, and only male employés were expected to turn out when the fire whistle blew. But Dr. Stewart, the medical superintendent of the hospital, has resolved that everyone connected with the hospital, from the house surgeon, through all grades, down to the kitchen maids, shall become expert in fire duty. The nurses are to learn how to use hook and axe, and they will be held responsible for the safe removal of patients in the case of a real fire. As promptness in answering the summons is one of the first essentials in a fireman—or fire-woman—it is doubtful if the management will introduce a special costume, but this addition to a nurse's work raises the question of the suitability of the present nursing uniform for such emergencies. The nurse who helps at a real fire should certainly not be clad in such inflammable materials as linen or cotton. Yet sanitary considerations forbid her wearing anything that is not strictly washable. Is it possible to render such materials thoroughly fireproof—that is, so that at the worst they will only singe and smoulder, without bursting into flame? If so, some such treatment, either in the original manufacture or in the regular laundrying, should be applied to the gowns, caps, and aprons of fire-girl nurses.

### NURSES AND DIPSOMANIA.

LAST week Dr. Robert Jones, medical superintendent of Claybury Asylum, lectured on "Dipsomania, its Causes and Cure," to the members of the Royal British Nurses' Association. Mr. Richard Greene presided, and there was a good attendance. Dipsomania, the lecturer said, was a disease which required nursing of the highest quality, and delicacy of touch in taking the pulse was of great importance. Some of the Claybury nurses were remarkable for the fineness of the tactile sense. The question of restraint was a very important one; an impulsive patient frequently tried to commit suicide, and it was the business of the nurse to remove, as far as possible, the means for accomplishing this purpose. She should see that the patient had a room downstairs. It was not absolutely necessary for such a patient to have two or three attendants, but numbers often prevented resistance. The tendency of a young nurse was to enter into struggles with refractory patients; this should be absolutely avoided. As far as possible, people should not be nursed in asylums. At the beginning they should be treated in their own homes, with the assistance of a trained hospital nurse. Tact and sympathy could not be overestimated. After giving some instructions relative to the use of drugs, the lecturer observed that the nurse should also understand massage; one of his nurses both used and taught it at Claybury. Finally, the nurse should exercise her ingenuity in finding suitable occupations for patients; these would prove a most useful tonic. With regard to "cures," hypnotism might be used with advantage in some cases, but always under medical supervision. The best working hypothesis was that based upon total abstinence.

### PROGRESS AT GATESHEAD.

It appears that in the North of England a ping-pong tournament has yielded even more to the Nursing Association than in the case of Southgate, which we recently mentioned. The amount realised by one of these entertainments at Gateshead was £87. This is a remarkable amount, and contrasts rather curiously with some other figures in the report. Thus, a Charity Cup football competition brought in £6 6s.; a Charity Cricket Match, £6 12s.; a glee party £5 2s. 6d. The smallest contributions should never be despised, but the popularity of ping-pong at Gateshead must be phenomenal. We are glad to learn that 1901 was the most successful year since the inauguration of the society, which, however, has to rely entirely upon the people themselves. No public body lends it a helping hand.

### HASTINGS NURSING ASSOCIATION.

In order to render the annual meeting of the Borough of Hastings and District Nursing Association on Monday next particularly attractive, the committee have invited Miss Constance Barker, lecturer to the Sussex County Council, to give an explanatory address, and are also arranging a musical entertainment of quite an elaborate character, among the performers being Miss Minnie Cochrane, lady-in-waiting to Princess Henry of Battenberg, an accomplished pianist. It has wisely been decided not to make any charge for admission.

### A HOME FOR FULHAM INFIRMARY NURSES.

It has been decided to erect a home for the accommodation of the nurses of Fulham Infirmary. The committee of guardians who were asked to report upon the subject visited the nurses' homes in connection with the unions of Brentford, Portsmouth, St. George's, Chelsea, Marylebone, St. Pancras, and Islington, the Poplar and Stepney Sick Asylum, and the London Hospital; and we are not surprised to learn that in all these places they "found the accommodation provided for nurses to be far in advance of that provided in the Fulham Infirmary." In accordance with their recommendation, the new home at Fulham is to consist of sufficient sleeping room for at least 45 nurses and an adequate staff of servants. There will also be dining-rooms, sitting-rooms, recreation-rooms, a lecture-room, and library. A block plan and an estimate of the total cost of the work are in course of preparation.

### AN INTERESTING AMERICAN ORGANISATION.

The Instructive Visiting-Nurse Association, in Baltimore, seems to be forging ahead. The annual meeting was held last month, and a paper having been read by Dr. Howard Kelly on "The True Spirit of the Nursing Profession," in which he dwelt upon the close relations existing between the medical profession and the organisation, other speakers referred to the valuable co-operation existing between the nurses and the district agents of the Charity Organisation Society, to the wide field open to the association in the treatment of tuberculosis in the homes of the poor, and to the need for a larger corps of nurses. After the speeches terminated pleasant social intercourse followed.

### HOSPITAL-TRAINED NURSES' INSTITUTE AT BEDFORD.

The Council of the Bedfordshire Hospital-Trained Nurses' Institute, which is not carried on with a view to pecuniary profit, but in order to maintain a high state of efficiency among the nurses, are so well satisfied that this object is carefully considered by the lady superintendent, that they have recently opened a nursing home for medical and surgical paying patients in a suite of rooms in a house adjoining the institute. As the patients are under a separate roof, the accommodation of the nurses in the institute is in no way curtailed, and their freedom, rest, and comfort, are not interfered with. The only financial result desired with respect to the new branch is that it should be self-supporting.

### NURSING SCARLET FEVER IN SHANGHAI.

From Shanghai we learn that nurses have been surrounded by scarlet fever cases. In the native city great havoc has been caused and hundreds of deaths have occurred. A servant taken to the isolation hospital died in two days; in one family of seven all died, and in another family five children. These are illustrations of the severe character of the epidemic. One of the great difficulties of nurses is to get the Chinese to understand what infection means. They talk about the disease, but will not take any precautions; and they do not think that they are responsible for anything that happens. The situation has been complicated by some of the natives falsely giving themselves out to be doctors and offering coloured water as an infallible cure for the disease. Worse still than this, others hawked about small tortoises, telling persons that if they had scarlet fever all they had to do was to hold a tortoise over their open mouth and squeeze its body so that a drop of liquid might fall from the creature's mouth down the infected throat and cure it!

### ST. LUKE'S HOSTEL.

At the annual meeting of St. Luke's Hostel last week, when the Archbishop of York presided, Dr. de Haviland Hall spoke in the highest terms of the nursing staff. Both matron and nurses were, he said, extremely able women, and all the patients had been unanimous in recognising their kindness and skill. The hostel is to be removed to more commodious premises as soon as a suitable site can be secured.

### SHORT ITEMS.

The King has conferred the decoration of the Royal Red Cross on Mrs. Osborn Howe in recognition of services rendered by her in attending to sick and wounded soldiers during the late war in South Africa.—The amount received during last quarter towards the fund for the Royal British Nurses' Settlement was £273 16s. 2d. The total amount now in hand is £2,645, of which £2,418 17s. 7d. is invested in trust stocks.—The Bishop of London will be one of the speakers at the annual general meeting of the Hammersmith and Fulham District Nursing Association on Tuesday evening next at 8 p.m.—Miss Florence Stokes, a St. Thomas's nurse, was married at Carew, Pembrokeshire, on the 19th instant, to Captain Francis Innis Day, Middlesex Regiment.

### Lectures to Nurses on Clinical Chemistry.

By CARSTAIRS DOUGLAS, M.D., B.Sc., F.R.S.Edin., Professor of Medical Jurisprudence and Hygiene, Anderson's College Medical School, and Dispensary Physician, Samaritan Hospital, Glasgow.

### LECTURE V.

THERE is only one other foreign body in urine with which we need concern ourselves, and that is pus. It may form a very considerable deposit, and when a patient shows this he is said to have "pyuria." As a rule, one relies on the microscope for the detection of pus, but there are two chemical tests that may be mentioned, as the means for performing them are found in all hospital test-rooms.

(1). The first is Donné's, to perform which the urine is poured off from the sediment and the latter in turn placed in a test-tube. To this, strong liquor potassæ is added, when the collection of pus is changed into a slimy, gelatinous, "ropy" mass, which can be poured as a continuous stream from one tube to another.

(2). Vitali's reaction. This is not often made use of. It illustrates one of the fallacies attendant upon the guaiac test for blood, mentioned in our last lecture. If some purulent urine be filtered, and, over the slimy residue which remains on the paper, a thin film of fresh tincture of guaiac be poured, a blue coloration will be noticed. That is to say, pus cells can oxidise guaiac to a blue tint without the intervention of ozonic ether. This test, as I have mentioned, is seldom made use of.

We have now considered the chemistry of urine as fully as is requisite for a nurse. I do not mean to say that we have by any means exhausted the subject, for there are many other bodies which we may look for, by chemical means, in the urine in disease, as, for example, acetone and diacetic acid in diabetes, fat in chyluria, hydrochinon in carbolic acid poisoning, and so on. But if a nurse knows how to examine a urine intelligently, as regards its physical properties and appearances, and how to test for albumin, sugar, blood, bile, and pus, she knows all that can be reasonably expected of her in this connection, and can be of the greatest help to the physician by properly applying that knowledge. Apart from the urine, clinical chemistry has not a very extensive field for its operations, though that field is a good deal wider now than it was, say, 15 years ago. It may be of interest if I indicate certain other lines in practical medicine along which clinical chemistry may proceed. It sometimes happens that a patient vomits small quantities of food some time after this has been swallowed, and the question may arise, Is this food returned from the gullet, either because there is a stricture at its lower end where it joins the stomach, and because it has a pouch (diverticulum) in its wall, or is it rejected actually from the stomach? That is to say, Has there been simply regurgitation of food, or true vomiting? Now we may find out a good deal about this matter by chemical means. Let us first of all take the reaction with litmus paper. If the material is alkaline or neutral, it has in all probability never been inside the stomach, but is still unaltered food mixed with saliva, which, as perhaps you know, is alkaline. But if the mass is distinctly acid, we suspect, and with good reason, that it has been in the stomach and has been mixed with acid gastric juice. We can still further examine it to see if the juice of the stomach has been acting on it, has in short peptonised it at all. To do this we test for peptones: we take a little of the fluid, add just a single drop of weak copper sulphate solution and then a good deal of liquor potassæ, when, in the presence of peptones, a delicate rose-pink colour appears. This is named the biuret reaction. In this way, you see, one can learn a good deal without much trouble.

More detailed examination of the gastric contents is often done nowadays in the wards for the sake of gaining information in cases of stomach disease. Most of the tests are applied to the discovery of the acids that may be present. Healthy gastric contents contain but one free acid—hydrochloric, a constituent of all normal stomach secretion. In many cases of cancer of the organ, this acid is secreted in very small amounts, and therefore in suspicious cases the acid is examined for, to determine its presence or absence. In not a few cases its place is taken by a new acid—lactic acid (which also occurs in sour milk), while in cases where the stomach is much dilated and the seat of fermentation of food, acetic and butyric acids may abound. The investigation of these acids is beyond a nurse's province, but there is no reason why she should not know that they exist and are sometimes sought for, and the purpose for which this is done.

Again, it may happen that a patient apparently coughs up some white frothy material, and examination of the chest reveals no disease, and suggests no cause why it should come from it. It is then thought that it may be fluid swallowed from the mouth, collected in some kind of pouch or recess in the upper part of the gullet, and at length ejected from this, much as if it had been coughed from the lungs. If this view be correct it should consist largely of swallowed saliva, and this is a substance that can be tested for with ease. For you may remember from your lectures on digestion that saliva contains a ferment named *ptyalin*, which can change starch into malt sugar; this, indeed, is the first digestive process in the body. If then we have a liquid which we suspect might be saliva we can try to find out if it has the power of altering starch in this way. Any nurse can do it. All that is required is to make a little weak solution of starch, by boiling a fragment in a little water in a test-tube, and, when it has cooled to blood heat, adding a little of the suspected fluid. If it be saliva that is present the ptyalin it contains will act quickly on the starch, converting it into malt sugar, and this can be easily tested for by Fehling's solution, just as we tested for sugar in urine. Neither saliva nor starch can reduce Fehling's solution, but malt sugar can and does. So you see that if a fluid coughed up from the chest has the power when added to starch solution of changing it into malt sugar which can reduce Fehling's solution, we are entitled to say that fluid consists in part or in whole of saliva.

I could multiply instances of the value of chemical methods in practical medicine still further, but neither time nor space will allow of this. They are applicable in the investigation of urinary and renal calculi of pathological fluids, *e.g.* from ascites, pleurisy or ovarian cysts, of the contents of the intestine, and so on. These matters, however, lie outside of the work of a nurse. Suffice it if she has a clear and adequate knowledge of ordinary urinary testing, and, in particular, a grasp of those principles upon which such methods rest.

### To Nurses.

WE invite contributions from any of our readers, and shall be glad to pay for "Notes on News from the Nursing World," or for articles describing nursing experiences, or dealing with any nursing question from an original point of view. The minimum payment for contributions is 5s., but we welcome interesting contributions of a column, or a page, in length. It may be added that notices of appointments, entertainments, presentations, and deaths are not paid for, but that we are always glad to receive them. All rejected manuscripts are returned in due course, and all payments for manuscripts used are made as early as possible after the beginning of each quarter

### The Nurses of the Central London Sick Asylum, Hendon.

INTERVIEW WITH THE MATRON. BY OUR COMMISSIONER.

THE lines of the nurses of the Central Sick Asylum at Hendon have fallen in pleasant places. The imposing range of buildings erected on the heights of North London by the Central London District Guardians for the reception of the sick poor supplies an excellent object lesson to all who desire to know how far it is possible for the Poor Law infirmaries to approach in construction and in equipment the great general hospitals which have been the pioneers of scientific treatment and trained nursing. Erected because the original institution in Cleveland Street, which, of course, still exists, was quite unequal to the strain imposed upon its resources, the site was wisely chosen with a view to fitness, to convenience, and to development. The surroundings, from the health standpoint, are all that could be desired; the infirmary is easily accessible from town, within a short walk or a shorter drive of the railway station, and only 6½ miles from the Marble Arch; and there is sufficient land adjoining for building purposes in the future. The building presents in appearance and arrangement a striking contrast to its parent institution, especially with regard to height. Except the front wings, the whole of the floors are in two stories, and as lifts are used for the meals there is therefore very little running up and down stairs. The corridors are heated by hot-water pipes and the electric light is made on the spot. A long inspection under the auspices of Miss Elma Smith, the matron, of the principal features, including some of the handsome and capacious wards, the excellent theatre, the useful dispensary, the remarkably large and admirably appointed laundry, and the comfortable nurses' home, justifies the opinion that the provision made for the patients and nurses at Hendon is of the most satisfactory character. Miss Smith has the great advantage of having been the head of the nursing staff at Cleveland Street, a post to which she was appointed after being trained at St. Bartholomew's Hospital, and has enjoyed experience as sister at York County Hospital, home sister at the Hospital for Sick Children in Great Ormond Street, and assistant matron at Paddington Workhouse Infirmary.

"When did you become matron in Cleveland Street?" I inquired.

"In 1892. At that time the period of training was two years. We increased it ultimately, about eight or nine years ago, to three."

### A Unique Institution.

"Then the three years' training has always been in force here?"

"Yes, it was decided upon about five years prior to the removal of the branch to Hendon. I came here as matron, and Miss C. Leigh was appointed matron in Cleveland Street."

"I conclude that the two institutions are absolutely separate?"

"Certainly, except that Dr. Hopkins is the medical superintendent of both. The whole of the patients are taken in, one by one, at Cleveland Street, on stretchers, and they are sent on in groups to Hendon by the medical superintendent, who arranges the ambulances. We are, I think, as an institution, quite unique."

### Differences in the Nursing Arrangements.

"Are the nursing arrangements identical?"

"Except in a few particulars; we have sisters here, and they have charge nurses there. This is because the nurses work under different conditions. Here a sister has charge of a block which consists of two large wards, and three, or four, or five small ones as the case may be. And we have an assistant matron, a night superintendent, four sisters, and 32 probationers in various stages of training."

"How does the number of beds compare?"

"There are 264 at Cleveland Street, and 331 at Hendon. Our four sisters have thus between them charge of the whole building. Each large ward has 32 beds, and the number in the small wards varies from three to five. You will judge that thoroughly capable women are a necessity here, in consequence of the skilful management needed with so many patients. Our assistant matron was night superintendent at Cleveland Street for several years, and each of the sisters has had valuable experience."

"Did any of the sisters come from Cleveland Street?"

"Two. We brought four probationers with us who were put on night duty, and the new probationers on day duty." All the others coming fresh at first to the work here.

### On and Off Duty.

"What are the hours on duty of the probationers?"

"They come on duty at 7 in the morning and go off at 8 30. The sisters come on and go off at the same time. We are very early in starting here. Breakfast, at which the assistant matron presides, is at 6.30. But there is a good deal of off duty. For instance, every member of the staff has three hours off at some period of the day, every Sunday. They also have half a day once a week, and an evening from 5 30 once a week. Then, they are often off duty from 1 until 4 30. Sisters and probationers have each three weeks' holiday. The differences as to the off duty are: that the sisters have a whole day off every month, and the probationers one every three months; the sisters also get 'scraps' of time which the probationers do not."

"How many probationers are on duty with the night superintendent?"

"One in each ward. No probationer is put on night duty for the first six months, and in the third year we call them staff nurses. I may say that we are never, under any circumstances, shorthanded. There are always extra nurses available when the work in the wards is heavier, or for assistance during holidays. I attach great importance to the maintenance at all times of an adequate staff. How can you go into a ward and complain that a thing is not being done properly, when there are obviously not enough people to do it?"

### Salaries and Instruction.

"What are the salaries of the sisters?"

"From £28 to £32 a year. There is no age limit. In fact, the more experience a sister has enjoyed the better."

"And the probationers?"

"They come at 21, and I do not care for them to exceed 30. I think that is a fair margin. It is, in my opinion, a great advantage that before a probationor enters, she should have learnt something about cookery, how to look after linen, and how to keep her cupboard neat. We pay the probationers £16, £17, and £18.

"Do you get a good class?"

"I think that, generally speaking, we get a very good class. I receive applications from all parts of the country, but, of course, there are only occasional vacancies. There may be some next May when our first final examination will take place."

"Is it open to the probationers to become sisters?"

"It will be if there are vacancies. But I would rather that when they are trained they should get fresh experi-

THE NURSES OF THE CENTRAL LONDON SICK ASYLUM, HENDON—*Continued.*

ence, and if they like, come back to us subsequently. We do as much as we can to help the staff nurses to understand sisters' duties. The senior duties are given them during the holidays, or in the absence of the sister, and if occasion requires, and we find some of them very adaptable. It is a reasonable inference that if they can take charge of a block here for three weeks, they may safely be recommended for other places."

"How soon do the probationers attend lectures?"

"Within six months. From October until May the medical superintendent and two assistant officers give lectures, and the matron and assistant matron hold classes every week. Also the sisters teach the juniors in the wards. There are examinations for juniors and seniors, who receive instruction separately."

THE NURSES' HOME.

"I noticed a number of cycles in the hall of the nurses' home. Are there other recreations?"

"There is tennis during the summer. It is quite easy to cycle to town, and many of the staff do so frequently. As you saw, the dining-room is in the administration block of the building, and all the meals are taken there."

"The Nurses' Home is completely shut off?"

"Yes, that was a part of the architect's plans. As a matter of fact, the nurses' home was opened at the same time as the asylum. The procession was formed there and walked up to the front door of the main building and opened it. In addition to the sitting-room for probationers and for sisters there is 'a quiet room' for writing or study."

"I see that each bedroom has a fireplace."

"And an easy chair. Each room has a fire in it once a week in cold weather. Quarters for sick nurses have not often been wanted, but there is accommodation for four invalids. If any nurse suffered from an infectious malady she would go into the isolation block."

"You have a fine large steam laundry."

"It is considered exceptionally good; but we need it, for between 19,000 and 20,000 articles have to be washed every week. We do the washing for Cleveland Street, and our own on alternate days in the week. All the drying as far as possible is done out of doors. There are 22 persons employed in the laundry, four of whom reside in the building."

THE "QUALIFED" NURSE QUESTION.

"Before I go, I should like to hear your views as to the proposal of the Departmental Committee to create "qualified nurses."

That is an arrangement with the authorities of the Local Government Board, and this institution is under their supervision. I have very decided opinions on the matter, but prefer not to express them.

### State Registration of Nurses: Some of its Difficulties.

BY A HOSPITAL SISTER.

STATE Registration of Nurses is a subject which has exercised the minds of most of us very much of late, especially since the State registration of midwives has become an accomplished fact. The frequent appearance of so-called "nurses" in the police courts on various disgraceful charges, naturally gives rise to great indignation in the nursing profession and makes its members desire some legal means of protecting its good name from the discredit cast on it by criminals, masquerading as "nurses," and using the uniform of an honourable profession as part of the stock-in-trade of their infamous callings, whether baby-farming, thieving, or worse. State registration seems at first sight to provide the needed weapon, and many of its advocates are led to desire it chiefly on that account.

THE ARGUMENT OF THE MIDWIVES ACT.

They argue, that as midwives are now to be registered, the necessary qualifications have been settled by law, and it is to be made penal for unqualified persons to practise, why should not nurses be registered, their qualifications settled, and their profession legally protected in the same way? They forget that "the Midwives Act" was passed to protect the public, especially the poor, from the danger of being attended by unqualified and ignorant persons. For the poor, as a rule, send for a midwife, not a doctor, who is not usually called in unless the case is a complicated one. A nurse is not an independent practitioner, but is usually sent for on the initiative of the doctor and works under his directions. The danger to the public, therefore, of untrained women nursing the sick is not such as to warrant compulsory registration. Nurses ask for State registration, not for the protection of the public, but for that of their own profession, and they think by means of State registration to organise a nursing body akin to the medical profession, to which only legally qualified women could be admitted. But if a Registration Bill is to be of use for the purpose desired, *i.e.* the prevention of unqualified persons practising for gain or posing as nurses for criminal ends, a clause making it penal for unqualified persons to practise, or to represent themselves as nurses, must form part of it, as in the Midwives Bill.

THE QUALIFICATION QUESTION.

The question of qualification seems to present insuperable difficulties. All sorts and kinds of attendance on the sick and feeble come under the general definition of "nursing"—from sitting up at night to keep in the fire and give drinks to a person with a cold in the head—to assisting at major operations and nursing cases of dangerous illness. It would be absurd to insist on three years' training as a minimum and to make it impossible for the general public to obtain the services of any but a three years' trained nurse, even for the most trivial ailments. Much of the "nursing" outside hospitals, whether in private houses or in district and cottage work, consists in making beds and washing helpless old people, which can be, and frequently is, perfectly well done by nurses with a very small amount of training; and it would be very cruel and unreasonable to deprive all these useful workers of their means of livelihood, unless it could be very clearly shown that they were a source of danger to the public. It stands to reason, that if it is to be made penal for unqualified women to nurse the sick and helpless, too high a qualification cannot be exacted either in the interests of the public or of the untrained, though often experienced, nurse, whose services the public need.

THE DANGER OF A LOW STANDARD.

Then on the other hand how would it improve the professional status of a three years' trained nurse to have her name on a State register where the names of nurses with two or one year's or even a shorter period of training were equally entitled to be found? A low standard would injure the profession and a high one be an injustice to the public. It might be suggested that there should be different grades of registered nurses, but how would it be possible to arrange by legislation what kind of case each grade would be qualified to undertake? Again, unless State registration is to be a farce altogether, some standard of training must be recognised, any and every "nursing home" or "hospital" could not be regarded as a training school. Probably the regulations for candidates for Local Government Board appointments would be adopted, and only nurses trained at a hospital of over a hundred beds would be eligible. If so, what would become of the smaller hospitals of which there are a vast number? It would be quite impossible for them to obtain probationers, as their training would not be recognised as qualifying their nurses for registration, and without registration nursing for pay would be illegal. The only other alternative to accepting every kind of institution, home, or cottage hospital as a training school, would be registration by examination regardless of previous training, which no sane person would consider for a moment as a test of a qualified nurse.

### The Dangerous "State Registered Nurse."

These difficulties do not seem to have been taken into consideration by the advocates of State registration for nurses, and one would like to know how they propose to overcome them. I should dearly like to protect our profession and uniform from being exploited by impostors, but I think that State registration would create more evils than it would remove, and when once any "hospital-trained" woman, however poor her training and qualifications, could be labelled State Registered Nurse, only gross misconduct could remove her name from the register, and as a "State Registered Nurse" she would be far more dangerous to her profession than any impostor who, after all, is very soon detected and exposed.

### Precautions in a Case of Fractured Pelvis.

### EXAMINATION QUESTIONS FOR NURSES.

THE question was as follows:—"Pending the doctor's arrival, what precautions should you take in a case of fractured pelvis, and in what manner should you place the patient in the bed?"

### First Prize.

In a case of pelvic fracture whilst waiting for the doctor's arrival I should tie the patient's knees together, and then with help very carefully place him on his back in a bed previously warmed, and with pillow and mackintosh under draw-sheet. I should raise the shoulders and place stiff bolster under knees. Avoiding the slightest unnecessary movement of patient I should take off or cut away his clothing and put on warm nightdress. Should it be a case of compound fracture I should place an antiseptic dressing on wound and fasten wide flannel binder round pelvis. Leaving one blanket next the patient I should then place cradle over injured parts, a hot-water bottle at the feet, and enough extra clothing to keep the patient warm and comfortable.

Should there be much collapse, and the doctor's arrival delayed, I should administer a little brandy and water.

I should then get catheters, probe, forceps, etc., all ready for use, also antiseptic lotions, dressings, etc., and hot water to hand.

I suppose it may be sometimes advisable for a patient to choose his own position, provided he keeps still. I remember a case in which the sacrum was fractured through the middle, and the patient could get relief from pain by lying only in a semi-prone position.—ELEANOR.

### Second Prize.

There is not very much can or need be done before the doctor's arrival. I should get the patient into bed by means of a flat board slipped underneath him, taking great care to keep patient flat on his back. Counteract shock or collapse by giving stimulant, extra blankets, and hot-water bottles in flannel covers. The comfort of the patient may be greatly promoted by applying a broad bandage as a support round hips; sandbags might be used if available. It will be remembered that the gravity is due more to the complications attending them than the injury to the bone. In case of the fracture having affected the pelvic organs, it would be necessary to see if patient passed urine after the accident, and notice if it contained blood.—HOPEFUL.

### Very few Candidates this Month.

The competition has been feeble both as to the numbers and the quality of the answers. It is true that the question is not a very easy one. Mercifully, cases of fractured pelvis are not often met with, consequently nurses' opportunities for observation are limited, but I advise that they should do their best to answer all questions. The object of our examinations is to increase knowledge, and therefore it is necessary for each one to see where she fails, or others do better. It would be very dull always to cling tenaciously to such well-beloved and time-honoured questions as those on the subject of bed-sores or the feeding of enteric cases. On these hundreds of nurses seem eager to express their views, but we must surely be a little more ambitious and occasionally strike into new ground.

### The Prize Winners.

"Eleanor" wins the first prize as the only candidate who mentions the highly important safeguard of tying the patient's knees together. This gives relief and effectually prevents further injury from the restlessness caused by great pain, when in desperation the sufferer is inclined to fling himself about as much as his disabled condition admits of. She also speaks of using a wide binder as a support to the pelvis. Both these measures are of the first importance. Thirdly, she shows knowledge of the subject when she says that it is sometimes advisable to allow a patient to choose his own position.

Several nurses have spoken of using long splints in such cases—they would be of little or no use, only touching at the outer edge of the hip-joint, and their action would be bad in slightly forcing the legs apart.

"Hopeful" mentions a board on which to move the patient to the bed—a most practical suggestion, as however carefully an injured person is moved in the recumbent position, there is a tendency to bend about the region of the pelvis. Later on this question will be repeated, it is hoped with larger results.

### Honourable Mention.

This is gained by "Nurse M. Rae," no pseudonym given; "Nurse L.", no proper name or pseudonym given; and "Caustic." The last-mentioned nurse is often successful.

### Question for February.

(1) If you had only 24 hours in which to prepare for an abdominal operation in a private house, what steps should you take with regard to the room to be used and the patient herself? (2) What preparations should you make to assist the surgeon to the best of your ability? It is presumed that he would bring all necessary dressings and antiseptic solutions.

THE EXAMINER.

### Charing Cross Hospital: The Nurses' Home.

THE handsome exterior of the new nurses' home at Charing Cross Hospital is not yet completed, and will not indeed be out of the hands of the builders for some little while, although the front entrance in Chandos Street presents a finished appearance to the passers by. Inside, however, there is not much more to be done, with the exception of rugs to be laid down, furniture to be got into place, and curtains to be arranged.

On Tuesday, through the courtesy of Miss Heather-Bigg, the new lady superintendent, who entered on her duties a fortnight ago, our representative was enabled to make a complete tour of the building. The new home is for the present reached by means of the female surgical and medical wards, because the covered passages, which on each floor will connect the hospital with the home, are not in a condition to be traversed. The first floor is exactly similar to the four floors above it except in regard to decoration. This has been kept distinct, the bedrooms on the first floor being a warm cream colour with doors, cupboards, and woodwork generally of a deep tone of pink termed *fraise écrasée*, similar to what used to be called simply "crushed strawberry." On the second floor a fresh bright green takes the place of the pink, the cream tint of the walls still prevailing. The third floor has blue for its distinguishing hue, whilst in the fourth a quiet sage-green is the dominant colour.

The number of bedrooms upon each floor is almost the same. The first has eleven nurses' and three sisters' rooms—of course every nurse has a room to herself—the second eleven nurses' rooms and rooms for two sisters and the out-patients' sister; the third, in addition to the nurses, accommodates two sisters and the theatre sister; and on the fourth floor, high above all noise and rush, is the bedroom for the night sister.

The rooms for the sisters differ in a few particulars only from those of the nurses. Every room has a fireplace, with a pretty painted overmantel, a bed, a washing-stand, a chest of drawers, a writing-table, a comfortable armchair, a bookcase, a cupboard for dresses and coats, and a reserve cupboard. The cupboard arrangements in particular are excellently planned. The doors fit very exactly, so as to prevent the entrance of dust; the brass hooks are four-sided, so as to receive four garments at once without one thing being put on the top of another; and there is ample room. Above this cupboard reaching up to the ceiling is another, which can be easily reached by a set of short steps with which every room will probably ultimately be fitted, or by standing on a chair. In this the nurses can store bonnet-boxes, unneeded clothes, and the numerous impedimenta which so often, for the want of such a convenient receptacle, have to lie about the room and tend to untidiness. The sisters' rooms are larger than the nurses', and instead of one window there are two. Also one of the cupboard doors is of plate glass, reaching down to the ground. These are the only distinguishing marks.

The furniture in every room is of "fume" wood, or wood which has been smoked till it has obtained a warm brown colour. The floors are polished, and the task of keeping them nice is to be given to the manufacturers of "Ronuk." There are two comfortable-looking rugs, which cover a good part of the floor, one of course being by the bedside. The electric light is laid on all over the home, and in the bedrooms by means of a long cord and-pulley it can be moved to any part of the room where it is most wanted. For fear at any time the current should suddenly fail, a second light supplied from a different source has been established on the other side of the room.

The lady superintendent's room, with bathroom beside it, is upon the first floor, the nurses' sick quarters upon the third. This is a large and pretty room, looking charming with its cream and blue walls, and though not as yet ready to receive patients, should be very cosy when fully furnished. Naturally, only non-infectious cases are to be treated here; any invalids who require isolation will be removed to the tower of the institution, where suspected cases amongst the inmates of the hospital are always sent. The fifth floor is to be occupied by the servants of the establishment, but this will probably be only a temporary arrangement. As the nursing staff increases—at present there are 10 sisters and 40 probationers—the other bedrooms may be required, and, as a matter of fact, the fifth floor is as well planned as the first, save that the roofs slope a little in odd corners.

The passages throughout the house are cream colour as to walls with terra-cotta dados, a line of bright blue marking the top of the dado. The floors are of a patent composition, specially made to deaden the sound of passing footsteps, and fire bells are placed at intervals, beside which stand pails of water and indiarubber hose. This can be screwed in a few seconds on to the pipes, connected directly with the main, and the chance of an accident through fire is accordingly lessened.

The sanitary arrangements on each floor are quite separate from the rest of the home, separate doors screen them off, and thus the drains are all outside the house itself and risk of infection is avoided. There are two lavatories and two bathrooms on each floor, and the baths are fitted with waste pipes which do their work in a very short time, so that no nurse need wait whilst the water runs away, but will find the bath ready for use directly she enters the room. An additional coil of hot water is placed in the bath corridor, so that there may be no striking and dangerous contact between the heated air of the bathroom and the cold passages. There is a lift for boxes, coals, etc.

Downstairs, on the ground floor, are the home rooms. These include a large handsome dining-room for the sisters, with solid mahogany chairs and tables. At one end of the room are doors which cut it off from the dining-room for the staff nurses and probationers. Both these rooms are decorated alike in a pleasing shade of green, and for festive occasions the door can be folded back and it can be made into one large room. This in its turn leads into the nurses' sitting-room, where the prevailing tints are peacock-blue and cream. Beyond is the library—a delightful room—the shelves of the bookcases well lined not only with volumes for recreation, but also with works of reference and instruction. The doctors of the hospital have taken great interest in contributing books which the nurses may find of value to them in their study. Next comes the sisters' sitting-room. Here, one side of the wall is made so that it can roll back on little wheels and double out of sight, thus converting two ordinary-sized rooms into one very large one. The lady superintendent's temporary sitting-room, which will hereafter belong to the staff nurses, is close at hand, and every one of the rooms on this floor can be made to communicate with each other so that an enormous reception could be held with ease upon occasions, and without the slightest overcrowding.

At present only breakfast and tea will be prepared in the home, dinner being sent over from the hospital kitchen. A large pantry, with a gas-stove, has been fitted up downstairs, where simple cooking can be done. The fixing of kitchen ranges has been deferred. The matron's permanent sitting-room and offices are yet to be built—they will form part of the new hospital—and as a chapel is a necessity and no upstairs room was available, religious services will for the time being be held in the basement, reminding one somewhat of the meetings of the Early Christians when, for purposes of secrecy, they were forced to worship in their cellars.

### Everybody's Opinion.

[Correspondence on all subjects is invited, but we cannot in any way be responsible for the opinions expressed by our correspondents. No communication can be entertained if the name and address of the correspondent are not given as a guarantee of good faith, but not necessarily for publication. All correspondents should write on one side of the paper only.]

### CIVIL HOSPITALS AND THE ARMY NURSING SERVICE RESERVE.

"W. S." writes:—May I ask Mr. Sydney Holland whether he thinks something should not be done by the authorities to help the nursing sisters of the A.N.S.R. who are now thrown out of employment by the boycott of the civil hospitals? Also whether he thinks it fair and right that those ladies who volunteered to go out to South Africa from patriotic feelings should be rewarded by being denied the employment for which they have gone through years of hard training and laborious work, besides having risked their lives in the service of their country?

### COST OF MAINTAINING A BICYCLE.

"CYCLIST" writes: I shall be very glad if some of your readers, who are district nurses and use bicycles, would kindly state in your columns what the annual cost is for repairs? I work with one other nurse in a wide parish, not very scattered, of 45,000 population, comprised almost entirely of working classes. The committee have bought one second-hand machine, and we have one of our own which we use; both are kept in repair by the association. We find that they cost together about £1 quarterly, which the committee seem to think too much, and I am anxious to know whether this is above the average amount.

### AIR-CUSHIONS.

"M. U. W." writes: May I be allowed to suggest to my fellow-nurses a sanitary method for filling air-cushions which I have found useful where bellows are not handy? Place your handkerchief single over the nozzle and blow steadily through this. The material does not impede the passage of the air, and the lips in this manner are not brought into contact with the metal.

### "THE PROBLEM SOLVED IN LANCASHIRE."

"IGNORAMUS" writes: I was very much surprised to read your note of last week headed, "The problem solved in Lancashire," about the arrangements in workhouse infirmaries in that part of the country. I am in a new workhouse infirmary in the Midlands, which contains over 500 beds. The night nurses are on duty for 12 consecutive hours with 60 patients to each nurse. The day nurses have on an average 30 to attend. The nurses' house is certainly separated, and is a very fine building; but the bedrooms are not properly heated, the temperature being 38°-40° F., and no fires are allowed. The mess-room is both bare and uninviting at any time. The wards are very fine, but badly arranged, and altogether it is a mismanaged institution, because there is no proper head; consequently no discipline is maintained. As this is my first Poor Law institution, and I have been informed that workhouses are all alike, you will understand my surprise.

### NURSES AT CONSUMPTION SANATORIA.

"ONE WHO WISHES TO KNOW" writes: Will you kindly allow me to ask whether it is usual in sanatoria for consumptives, for the nurses to eat and drink from the plates and glasses, etc., used by the patients? It seems to me that glasses, and cups particularly, used many times daily by bad cases, and only roughly rinsed, must be the source of possible infection. Also is it perfectly wholesome to use the excreta from phthisical patients as manure for the cabbages in the kitchen garden?

### Appointments.

[No charge is made for announcements under this head, and we are always glad to receive, and publish, appointments. But it is essential that in all cases the school of training should be given.]

CYMMER ISOLATION HOSPITAL, PORT TALBOT.—Miss May Davies has been appointed nurse-in-charge. She was trained at Brixham Hospital, and was afterwards in charge of the Royal Victoria Homes Infirmary. For the last two years she has been assistant nurse at the City Hospital, Ham Green, Bristol.

EAST LONDON HOSPITAL, SHADWELL, E.—Miss Georgina Bamber has been appointed night sister. She was trained at the Royal Hospital for Sick Children, Glasgow, and has since been ward sister at the Children's Infirmary, Liverpool.

ESTON URBAN DISTRICT SANATORIUM.—Miss Jeannette Frood has been appointed matron. She was trained at the Dumfries and Galloway Royal Infirmary, and was afterwards charge nurse at the male wards, Smithston Infirmary, Greenock, N.B.; on the staff of the Oldham Nursing Association, charge nurse at Leeds Union Infirmary, sister-in-charge at the North Riding Infirmary, Middlesbrough, and superintendent nurse at the Union Hospital, Bishop Auckland.

GOVERNMENT ASYLUM FOR INSANE, ST. JOHN'S, NEWFOUNDLAND.—Miss Catharine Fraser has been appointed matron. She was trained and held the post of charge nurse at Morningside Asylum, Edinburgh.

GOVERNMENT CIVIL HOSPITAL, MAURITIUS.—Miss Helen Batchelor has been appointed matron. She was trained at the London Hospital for three years and took the L.O.S. certificate after special training at the Clapham Maternity Hospital. She has worked for the Government at the Hong Kong Civil Hospital and at Wei-hai-wei during the late disturbances in China. During the last year she has held matron's posts in the refugee camps in the Orange River Colony.

INFANT ORPHAN ASYLUM, WANSTEAD.—Miss H. N. Rutt. has been appointed sister-in-charge. She was trained at the Leicester Infirmary, and was afterwards theatre sister at Lewisham Infirmary, sister at the Great Northern Central Hospital, and sister at the East London Hospital for Children Shadwell.

INCORPORATION INFIRMARY, SHIRLEY WARREN, SOUTHAMPTON.—Miss M. Cicely Elmhirst has been appointed sister. She was trained for two years at the Royal Alexandra Hospital, Rhyl, and was attached to the private staff of that institution until 1898. She afterwards joined the private staff of Princess Christian's Nurses' Home at Windsor. From 1899 to 1902 she received training at Chelsea Workhouse Infirmary.

ISOLATION HOSPITAL, WATFORD.—Miss Mary Elliott has been appointed matron. She was trained at St. Thomas's Hospital, London, and was afterwards nurse for five years. Later she was day sister and night superintendent at the Monsall Fever Hospital, and matron of the Blackburn Fever Hospital.

LODGE MOORE HOSPITAL, SHEFFIELD.—Miss Catherine Mairi Duffy has been appointed night superintendent. She was trained at the Royal Albert Edward Infirmary, Wigan, and was for two years sister at the Parkhill Hospital, Liverpool.

QUEEN ALEXANDRA'S IMPERIAL MILITARY NURSING SERVICE.—Miss Beatrice Isabel Jones has been appointed matron at the Herbert Hospital, Woolwich. She was trained at St. Bartholomew's Hospital, London, and has since been assistant matron at Birmingham Infirmary and matron of the City of London Hospital for Diseases of the Chest. She has also served in South Africa as a member of the Army Nursing Service Reserve.

QUEEN CHARLOTTE'S LYING-IN HOSPITAL, MARYLEBONE ROAD, N.W.—Miss Kate Spencer has been appointed superintendent of the Nurses' Home of Queen Charlotte's Hospital. She was trained at the London Hospital, was subsequently home sister at the Poplar Hospital, and has recently been holding the post of matron of the Suffolk Convalescent Home at Felixstowe.

THAMES DITTON COTTAGE HOSPITAL.—Miss Louise M. de Voute has been appointed staff nurse. She was trained at Watford Cottage Hospital, and has since been staff nurse at Woolwich and Plumstead Cottage Hospital.

### Presentations.

CITY OF GLASGOW FEVER HOSPITAL.—On the occasion of leaving Ruchill Hospital, Glasgow, to become matron of the McKelvie Hospital, Oban, Miss Agnes Espie, one of the assistant matrons, received the following presents:—From a number of the nurses a china afternoon tea-set, from the maids a beautiful morocco leather dressing-case, and from the men a handsome case of silver afternoon-tea and jelly spoons and butter-knife—also a silver and crystal jelly-dish, besides numerous other presents—as tokens of their appreciation of her kindness and consideration for them.

### The Nurses' Bookshelf.

"THE NURSING PROFESSION: HOW AND WHERE TO TRAIN." By Sir HENRY BURDETT, K.C.B. (London: The Scientific Press. Price 1s. 6d.)

THIS convenient volume has now reached its fourth year of publication, and is increasingly in demand among women who desire to become nurses. Whereas formerly matrons and secretaries were compelled to answer at length the queries, many of them irrelevant, of aspirants, they can now simply refer them to Sir Henry Burdett's volume, wherein they will find all the information which they can possibly need to enable them to make choice of a training school. They will find the details given for every hospital in the kingdom and many in the Colonies, India, and the United States, as to the length of training given, the limits of age within which probationers are received, the premium demanded, and the salary given. In a preliminary chapter is given a summary of the duties required from probationers, nurses, and sisters, a knowledge of which would often save a well-meaning but incompetent girl from trying to enter a profession for which she is unfitted. But it is not to intending nurses alone that this volume is of use. Those who have completed their training, especially if they are engaged in private work, will be very grateful for the summary of the principal laws affecting nurses which is here given. They will find guidance as to their duties to their patients, their rights as to salary, engagement, and dismissal, and their responsibilities towards medical men and the law of the land. From ignorance of these things many nurses suffer injustice and others receive undeserved blame. A study of the chapter to which we refer would often save both of these much trouble and inconvenience. In short, this volume, interesting, cheap, and convenient, should be in the hands of every woman who is, has been, or hopes to be a nurse. Besides the information as to training schools, the book contains many useful details of the Royal National Pension Fund, and other provident and charitable funds for nurses, and the distinctions for which they are eligible.

### The British Nurses' Association and the "Qualified Nurse."

THE following memorial has been addressed by the Royal British Nurses' Association to the Right Honourable Walter Long, M.P., President of the Local Government Board:—

SIR,—At a meeting of the General Council of the Royal British Nurses' Association held on Friday, January 23rd, it was unanimously resolved to most respectfully call your attention to a report which has been extensively circulated to the effect that it is the intention of the Local Government Board to grant certificates to nurses who have had one year's training in certain Poor Law Institutions, and that this certificate shall entitle the holder to describe herself as a "qualified nurse." The Royal British Nurses' Association, which was founded in 1887, and was incorporated by Royal Charter in 1893, under the distinguished presidency of her Royal Highness the Princess Christian, has sought from the beginning to promote the thorough training of nurses as essential to the interests of the sick people who come under their care; and it has laid down three years as the minimum amount of training which a nurse should receive before she is entitled to be regarded as a "qualified nurse," which standard has been almost universally adopted at all the great hospitals where large numbers of nurses are trained, and is now rapidly extending to the more important hospitals in the British Colonies. This Association would most respectfully urge that a certificate entitling the holder to style herself a "qualified nurse" after only one year's training issued by such an important department of the State as the Local Government Board would very seriously injure their endeavours and those of others to raise the standard of the education and training of qualified nurses. The Association is much interested in the steps which are being taken by the Local Government Board to organise and improve the nursing arrangements in the Poor Law institutions, more especially in those situated in some of the more remote parts of the country; it is also very sensible of the difficulties that stand in the way of organising a satisfactory system of nursing under conditions that must necessarily vary from time to time. Whilst entirely sympathising with the objects of the Local Government Board the Association would suggest the adoption of a nomenclature which is not liable to be misunderstood. If it is thought that with regard to the nurses who commence their training in the minor training schools the term "probationer" should not be retained until they are qualified to pass into a superior class, it is suggested that the term "staff nurse" might be employed to designate those probationer nurses who have had at least one year's, and less than three years' training, and that this would prevent any misconception as to their status, and also possible abuse of the term "qualified nurse."

Signed on behalf of the General Council,

HELENA (President).

### Where to Go.

THE LONDON GLOVE COMPANY.—This establishment will start its winter sale on Monday, February 2nd, at 45A Cheapside, E.C.

### Death in Our Ranks.

WE regret to announce the sudden death on January 22nd of Nurse Annie Bryant, of the Mildmay Nurses' Home, 10 Newington Green. Miss Bryant had been a member of the private staff for eight years, and was much beloved by all who knew her.

### Echoes from the Outside World.

### The Royal Christening.

THE baptism of the infant son of the Prince and Princess of Wales took place in the private chapel at Windsor Castle on Monday afternoon. Flowers from the King's conservatories adorned the building, and the Queen placed the royal child in the arms of the Bishop of Oxford, who performed the baptismal rite. The sponsors were the King, Prince Waldemar of Denmark — represented by Prince Charles of Denmark—and Prince Louis of Battenberg, godfathers; the Queen, the Empress of Russia—represented by Princess Victoria—and Princess Christian of Schleswig-Holstein, godmothers. The Prince received the names of George Edward Alexander Edmund. After the ceremony the company were entertained by their Majesties, the royal party lunching in the Oak Room and the rest of the visitors in the State dining-room. In the evening the King, to mark the christening of his grandchild, gave a large dinner party in the Waterloo Chamber, to which a number of guests were invited. After dinner the Master of the Household gave the toasts of "Their Majesties the King and Queen" and "Prince George Edward Alexander Edmund of Wales."

### Mr. Chamberlain and the Boer Enthusiasts.

AFTER Mr. Chamberlain left Johannesburg, last week, he paid a visit to Potchefstroom, the old capital of the Transvaal, and inspected the British burgher settlement. But the most interesting incident in this connection was the appearance of the Colonial Secretary at the farm of General Andries Cronje, who commanded the first wing of the National Scouts. All the settlers in the district had assembled at the farm, as well as several Boers from the outlying parts. Even more remarkable than the triumphal arches and decorations was the enthusiasm manifested by some of the Boers, who removed the horse from Mr. Chamberlain's carriage and dragged the vehicle past the long line of wagons outspanned along the main approach. Mr. Chamberlain, in reply to an address of welcome, expressed, amid great cheering, the hope that many of those who were *bywoners* in the past, without land of their own, would now have an opportunity of keeping themselves and their children. On Monday the Colonial Secretary visited Lechtenburg, and his acknowledgment of an address was followed by a speech from General Delarey. Both speakers were loudly cheered by the burghers. On Tuesday "gallant little Mafeking" had the satisfaction of welcoming Mr. Chamberlain's party.

### A Member of Parliament Sentenced to Death.

NOTHING in the trial of "Colonel" Lynch, which terminated on Friday, caused so much astonishment at the moment as the finale. And yet there was no other sentence possible than that of death, for there was abundant proof forthcoming that the charge of high treason, of which the prisoner was accused, was well proved, and the punishment for high treason is death. "Colonel" Lynch is by birth a British subject. After the war broke out he went to the Transvaal as correspondent to a Paris journal, got himself naturalised as a burgher of the Transvaal State, and made a declaration renouncing all his former rights. On the same day he took the oath of allegiance to the Republic, then at war with Great Britain. He issued an address to Irishmen urging them to be "up and doing," and accepted the command of a brigade composed of all nationalities, described as "the Irish Brigade." At Glencoe and elsewhere, he shared in the fighting against the British troops, recruited forces, and commandeered supplies, and, it is said, showed himself particularly anxious to have two men shot, because, he alleged, they were British spies. During his absence in Paris on his return from South Africa he offered himself for a British constituency, Galway, in Ireland, and was elected in his absence as a Member of Parliament. When he landed in England he was promptly arrested, and has already been in gaol for nearly a year. In passing sentence, Mr. Justice Wills, who, like the Lord Chief Justice and Mr. Justice Channell, wore a black cap, omitted the intimation that the body was to be "drawn and quartered," and on Wednesday it was announced that the sentence had been commuted to penal servitude for life.

### A Lady Poor Law Commissioner in the West Indies.

AT the instance of the Governor of the Bahamas, Mrs. Anton Bertram has been appointed one of the three commissioners of the New Providence Asylum, who have full charge of the whole Poor Law system of the colony. Mrs. Bertram's husband is Attorney-General for the Colony, and before her marriage she was, as Miss Edith Rees Jones, a well-known member of the Cardiff Board of Guardians. As recently as last year she attended the conference of Poor Law Unions in London as one of the representatives of Cardiff, and she was also a speaker at the annual meeting of the State Children's Association.

### The Law's Delay.

ON Saturday the second trial of William Gardiner for the wilful murder of Rose Harsent, a servant girl at Peasenhall, in Suffolk, was concluded at Ipswich. The first trial took place 234 days ago at Bury St. Edmund's, with the result that the jury could not agree. The issue at the Ipswich trial was the same, and the prisoner has now to be tried a third time, in June, at Bury St. Edmund's. This means that it will be some five months before he is put out of his suspense, and the scandal of such a state of things threatens to lead to an agitation for the adoption of the Scotch law which allows a verdict of "Not proven."

### The New Savoy Opera.

A NEW musical piece, entitled "A Princess of Kensington," was produced at the Savoy Theatre last week. Captain Basil Hood is responsible for the libretto, and there is much in it which is quaint and original. The music, written by Mr. Edward German, has several numbers which are likely to become popular at once. Kenna, Oberon's daughter—from whom Kensington is said to be named—was engaged, a thousand years ago, to Azuriel, a stalwart fairy, but jilted him for Prince Albion, a mortal. But Albion was put to sleep by a yellow fog which Neptune sent, and now the time of his awaking is near at hand, and Azuriel, to whom Kenna's love has returned, refuses to have anything to do with her till Albion is safely wedded to someone else. Puck undertakes to find a wife for Albion before sunset, and it is the various difficulties that arise in consequence of this undertaking which create so much wholesome pleasant merriment. The first scene takes place in Kensington Gardens, and a very pretty picture is made by a faithful representation of the well-known spot. The most remarkable success was made by Miss Constance Drever, a young lady who, at two days' notice, accepted the part of the heroine Kenna, and played it exceedingly well. Miss Brandram as the niece of the landlord of the "Jolly Tar," is very diverting, Mr. Evett sings delightfully as Brook Green, Miss Winifred Hart-Dyke's dancing is perfect, and a large meed of praise must be accorded to Mr. Henry Lytton.

### For Reading to the Sick.

THE MOUNT OF OLIVES.

AH! methinks I see Thee now
Climbing, late, the mountain side;
Cool night-breezes fan Thy brow,
Day's long cares in shadows hide:
Far below the eastern steep
Salem lies in double sleep.

Nay, He rests not, see Him there
Kneeling low upon the sod,
All the burden of His prayer
Pouring forth as man to God,
Far away from earthly jars
In the clear calm light of stars.

But the first grey light of morning
Pierces now the olive shade;
Early birds, with gentle warning,
Carol through the leafy glade:
All unrested, save by prayer,
Jesus drinks the morning air.

. . . . .

Then, when that dark eventide
Closes in our life's long day,
And, like some steep mountain side,
Frowns the last and lonesome way,
Bright to us that path shall be,
Found alone, O Lord, with Thee!

*C. L. Ford.*

In the dim evening light, a picture of the darkness that was coming over His soul, He goes forth to prepare Himself for His pain by earnest prayer. See Him kneeling alone in the garden cave, His "soul sorrowful unto death." And yet the same prayer was uttered, without variation, nay, with an ever-increasing earnestness: "Father, if it be possible, let this cup pass from Me; yet not My will but Thine be done."

"Let patience have her perfect work, that ye may be perfect and entire, wanting nothing" (St. James i. 4). But the great end will be promoted chiefly by our ever seeking more and more of His Life in ourselves, through the indwelling of His Spirit; "till we all come, in the unity of the Faith, and of the knowledge of the Son of God, to the Perfect Man; to the measure of the stature of the fulness of Christ."

The future is not clear to you as it was to our Lord; you live only from day to day, and often "the chalice" presented to you seems bitter to drink, especially when you see no result to your pain. Often, probably, the thought comes to you that you are not wanted, that you are unable to do any good in the world. Unite yourself at such times with our Lord at His agony; speak out the feelings of your heart fully and naturally to your heavenly Father; pray for relief, pray for strength; and, cheered by the vision of our Lord's great victory over sin, accomplished by so much pain, let your prayer be the same as His, resting assured that your pain, even as His, is bearing fruit, though you see it not. And your Angel Guardian shall bring to you "a chalice" from above, mixed by your Father and truest Friend, "containing in itself all sweetness," for, though it be bitter to the taste, it is sent for the glory of God and the good of your soul.—*Anon.*

### Notes and Queries.

**The Editor is always willing to answer in this column, without any fee, all reasonable questions, as soon as possible.**

**But the following rules must be carefully observed:—**

1. **Every communication must be accompanied by the name and address of the writer.**
2. **The question must always bear upon nursing, directly or indirectly.**

**If an answer is required by letter a fee of half-a-crown must be enclosed with the note containing the inquiry, and we cannot undertake to forward letters addressed to correspondents making inquiries. It is therefore requested that our readers will not enclose either a stamp or a stamped envelope.**

*America.*

(128) I should be glad if you would tell me the best way to get an appointment in an American hospital. Can you kindly give me the addresses of any?—*L. M. F.*

You will find the addresses and some particulars of the principal American hospitals in "Burdett's Hospitals and Charities." But there are plenty of American trained nurses to fill all vacancies many times over, and the foreigner has a hard battle to fight to win recognition.

*Daily Nursing.*

(129) Will you inform me if there is an association in London for employing nurses daily for the middle classes?—*S. M. H.*

The Marylebone Daily Visiting Nursing Association. Mrs. Spring Rice, 1 Bryanston Place, W., will supply any details.

*Massage.*

(130) Will you tell me where I could learn massage thoroughly and the probable cost, also what books I should read up in the meantime?—*W. S. F.*

The National Hospital for the Paralysed and Epileptic, Queen's Square, Bloomsbury. The fees vary from £10 10s. to £40, according to the term of training. You might also write to the Secretary of the Society of Trained Masseuses, 12 Buckingham Street, Strand, W.C.

*Maternity.*

(131) A nurse, with 12 months' hospital training, is anxious to take the best maternity training. She would like to know if there is a certainty of work after training. Can anyone advise her?—*Nurse H.*

There can never be a certainty of work. A good maternity nurse has a fair prospect of making a living. See other queries under this heading, as they may probably help you.

*Davos.*

(132) Can you tell me if there is a sanatorium for the treatment of consumptives at Davos, under the direction of English people? I shall be glad of any information.—*J. Von B.*

The Davos Invalids' Home, Davos Dorf, is a well-known institution. Dr. W. Ewart, 33 Curzon Street, W., is the Hon. Secretary.

*Cannes.*

(133) Will you kindly give me the address of nursing institutes in Cannes, and say if nurses going there would be likely to obtain private work without joining an institution?—*Nurse S.*

The nearest nursing institute to Cannes is the Nice Nursing Institute, Villa Pilatte, Avenue Désambrois, Nice. It is possible that you might obtain private work, but of course there is always the risk of success.

*Death Certificate.*

(134) Can a midwife give a certificate of death for a still-born baby when there is a doctor at hand?—*L. R.*

Certainly not.

**Standard Nursing Manuals.**

**"The Nursing Profession: How and Where to Train." 2s. net; post free 2s. 4d.**
**"A Handbook for Nurses." (Illustrated). 5s.**
**"Nursing: Its Theory and Practice." (New Edition.) 3s. 6d.**
**"Nursing in Diseases of Throat, Nose, and Ear." 2s. 6d.**
**"Surgical Ward Work and Nursing." (Revised Edition.) 3s. 6d. net; post free, 3s. 10d.**
**"Art of Massage." (Second Edition.) 6s.**
**"Elementary Physiology for Nurses." 2s.**
**"Elementary Anatomy and Surgery for Nurses." 2s. 6d.**
**"Practical Handbook of Midwifery." 6s.**
**"Mental Nursing." 1s.**
**"Art of Feeding the Invalid." 1s. 6d.**
**All these are published by the SCIENTIFIC PRESS, Ltd., and may be obtained through any bookseller or direct from the publisher. 28 and 29 Southampton Street, London, W.C.**

